# In Vivo Choroidal Vascular Lesions in Diabetes on Swept-Source Optical Coherence Tomography

**DOI:** 10.1371/journal.pone.0160317

**Published:** 2016-08-01

**Authors:** Tomoaki Murakami, Akihito Uji, Kiyoshi Suzuma, Yoko Dodo, Shin Yoshitake, Rima Ghashut, Rina Yoza, Masahiro Fujimoto, Nagahisa Yoshimura

**Affiliations:** Department of Ophthalmology and Visual Sciences, Kyoto University Graduate School of Medicine, Kyoto, Japan; University of Michigan, UNITED STATES

## Abstract

Diabetes induces microvascular diseases including diabetic retinopathy and choroidopathy which reciprocally promote the pathogenesis, although optical coherence tomography images of diabetic choroidopathy remains to be documented. Here we evaluated the qualitative characteristics of choroidal vascular lesions in patients with diabetes and their association with diabetic retinopathy on swept-source optical coherence tomography (SS-OCT) images. We retrospectively reviewed 110 consecutive eyes of 110 patients with diabetes and 35 eyes of 35 healthy subjects for whom SS-OCT images (6x6-mm scans centered on the fovea) of sufficient quality were acquired. The curve of chorioretinal sections was flattened using Bruch’s membrane as a reference surface, followed by generation of en-face images. We characterized choroidal vascular lesions and evaluated their association with the logarithm of the minimum angle of resolution visual acuity (logMAR VA), retinal and choroidal thicknesses, and diabetic retinopathy severity. En-face SS-OCT images showed unvisualized vessels in Sattler’s layer in 33 eyes (30.0%). Focal narrowing was seen in choroidal vessels in Haller’s layer in 56 eyes (50.9%). The choroidal vessels ended in the superficial or middle portion of Haller’s layer, referred to as vascular stumps, in 20 eyes (18.2%). Diabetic eyes had these findings more frequently than nondiabetic eyes. The subfoveal choroid was thicker in eyes with focal vascular narrowing and vascular stumps than in eyes without such lesions. Vascular stumps in Haller’s layer were significantly related to diabetic retinopathy severity, logMAR VA, and central retinal and choroidal thicknesses. These novel findings on SS-OCT images would promote the better understanding of complicated pathogenesis in diabetic retinopathy and choroidopathy.

## Introduction

Diabetic retinopathy (DR), a diabetic microangiopathy, leads to severe visual impairment[1, 2]. Despite treatments including anti-vascular endothelial growth factor (VEGF) therapy, patients sometimes have poor visual prognosis and the pathogenesis in chorioretinal vasculature remains to be further elucidated[3–5]. Diabetes induces macrovascular and microvascular complications systemically, and a few histologic publications have proposed “diabetic choroidopathy” as another diabetic ocular complication[6–8].

The choroid is comprised of stroma and vasculature which the posterior ciliary arteries feed and vortex veins drain. The choroidal vessels with a larger or smaller diameter are mainly in Haller’s and Sattler’s layers, respectively, and further branch off to the choriocapillaries, which are in contact with Bruch’s membrane and substantially nourish the outer retinal layers through the retinal pigment epithelium[9]. Previous histologic publications have reported thickening of the vascular basement membrane, arteriosclerotic changes, or vascular luminal narrowing in the choroid of patients with diabetes[6]. Capillary dropout and leakage of proteinaceous fluid into the stroma also have been reported and may represent ischemia and vascular hyperpermeability, as clinically shown in the retinal vasculature in DR[7]. Recent research has suggested a definite damages in the photoreceptor cells in DR[10, 11], although it remains ill-defined how diabetic choroidopathy affects the pathophysiology in the outer retinal layers.

Enhanced depth imaging of spectral-domain optical coherence tomography (OCT), facilitates measurement of the choroidal thickness and elucidates changes in the choroidal vessels in several chorioretinal diseases[12]. Many researchers have evaluated the choroidal thickness in patients with diabetes and reported associations with DR severity, diabetic macular edema (DME), or foveal morphologic patterns[13–18]. Several publications have reported a relationship between the choroidal thickness and the visual prognoses after medical interventions for DME[19–22]. It leads us to consider whether morphologic or functional changes in the choroidal vasculature affect the choroidal thickness and concomitant retinal dysfunction in DR.

Recent advances in swept-source (SS)-OCT, i.e., a longer wavelength, faster scanning speed, and invisible scanning light, have achieved better three-dimensional choroidal images[23], which allow us to better evaluate choroidal vascular changes in Sattler’s and Haller’s layers. In this study, we investigated the morphologic findings in the choroidal vasculature and their associations with choroidal thicknesses and other DR parameters in patients with diabetes.

## Materials and Methods

### Patients

We retrospectively reviewed 110 consecutive eyes of 110 patients with diabetes on whom SS-OCT images of sufficient quality were obtained (Table 1). Four patients had type 1 diabetes mellitus (DM) and 106 had type 2 DM, specifically, 27 eyes with no apparent retinopathy, eight with mild nonproliferative diabetic retinopathy (NPDR), 31 with moderate NPDR, 10 with severe NPDR, and 34 with proliferative diabetic retinopathy (PDR), who visited the Department of Ophthalmology of Kyoto University Hospital from February 2014 to August 2014. The eyes of patients with diabetes were included for which SS-OCT images of sufficient quality were acquired. We selected right eyes, and if right eyes had poor quality of SS-OCT images or met the exclusion criteria below, left eyes were evaluated. The exclusion criteria were the presence of other chorioretinal diseases, high myopia, glaucoma, or ocular hypertension; a history of any intervention for macular lesions including anti-VEGF therapy and intravitreal or sub-Tenon’s administration of steroids within 6 months; photocoagulation in the macular area; intraocular surgery other than cataract extraction; and cataract surgery within 6 months. We also excluded eyes in which severe intraretinal lesions including retinal hemorrhages and hard exudates were an obstacle to choroidal imaging or those in which the images had errors in the automatic segmentation of Bruch’s membrane. We selected unaffected fellow eyes of age- and hypertension-matched retinal vein occlusion patients as nondiabetic control subjects, and the same exclusion criteria were applied to these control patients (Table 1). We consecutively screened 228 diabetic eyes and 60 nondiabetic control eyes, and excluded 118 diabetic eyes and 25 control eyes according to these criteria. All research and measurements adhered to the tenets of the Declaration of Helsinki. The institutional review board and the ethics committee of Kyoto University Graduate School of Medicine approved the study protocol. All participants provided written informed consent.

**Table 1:** Choroidal Vascular Changes in Control Subjects and Diabetic Patients.

	Control subjects (n = 35)	Diabetic patients (n = 110)	*P* Value
Age (years)	63.4±12.6	62.9±13.5	0.832
Gender (male/female)	14/21	67/43	0.034
HbA1c	-	7.67±1.43	-
Hypertension (present/absent)	21/14	66/44	1.000
Retinal thickness in the central subfield (μm)	239±23	268±86	0.055
Subfoveal choroidal thickness (μm)	226±68	244±77	0.223
Unvisualized vessels in Sattler’s layer(present/absent)	4/31	33/77	0.028
Focal vascular narrowing in Haller’s layer (present/absent)	6/29	56/54	<0.001
Vascular stump in Haller’s layer(present/absent)	1/34	20/90	0.026
Aneurysmal changes in Haller’s layer(present/absent)	6/29	28/82	0.367

### Swept-Source OCT

After a comprehensive ophthalmic examination, we acquired three-dimensional volumetric datasets from the vitreous cavity to the sclera in the macula using SS-OCT (DRI OCT-1, Topcon, Tokyo, Japan). This system acquires 100,000 A-scans/sec at wavelength of 1050 nm with band width of ~100 nm. It allows us to acquire the images of uniform quality over depth, because of better penetration with less scatter. In addition, invisible scan lines and eye tracking system further improve the continuity between B-scan images and reduce the lateral displacement in the volumetric images. To correct the horizontal scan length, we first input the axial lengths that were measured using partial coherence interferometry (IOLMaster, Carl Zeiss AG, Oberkochen, Germany). Raster scans (6 x 6 mm) centered on the fovea comprised of 256 horizontal B-scans generated from 512 A-scans were obtained using the 3D scan mode. The raw data were exported through the OCT Viewer (Topcon), followed by further image processing. Subfoveal choroidal thickness (the mean thickness from the retinal pigment epithelium / Bruch’s membrane complex to the sclerochoroidal interface within central 1 mm) was measured using the manufacturer’s software.

**Fig 1:**
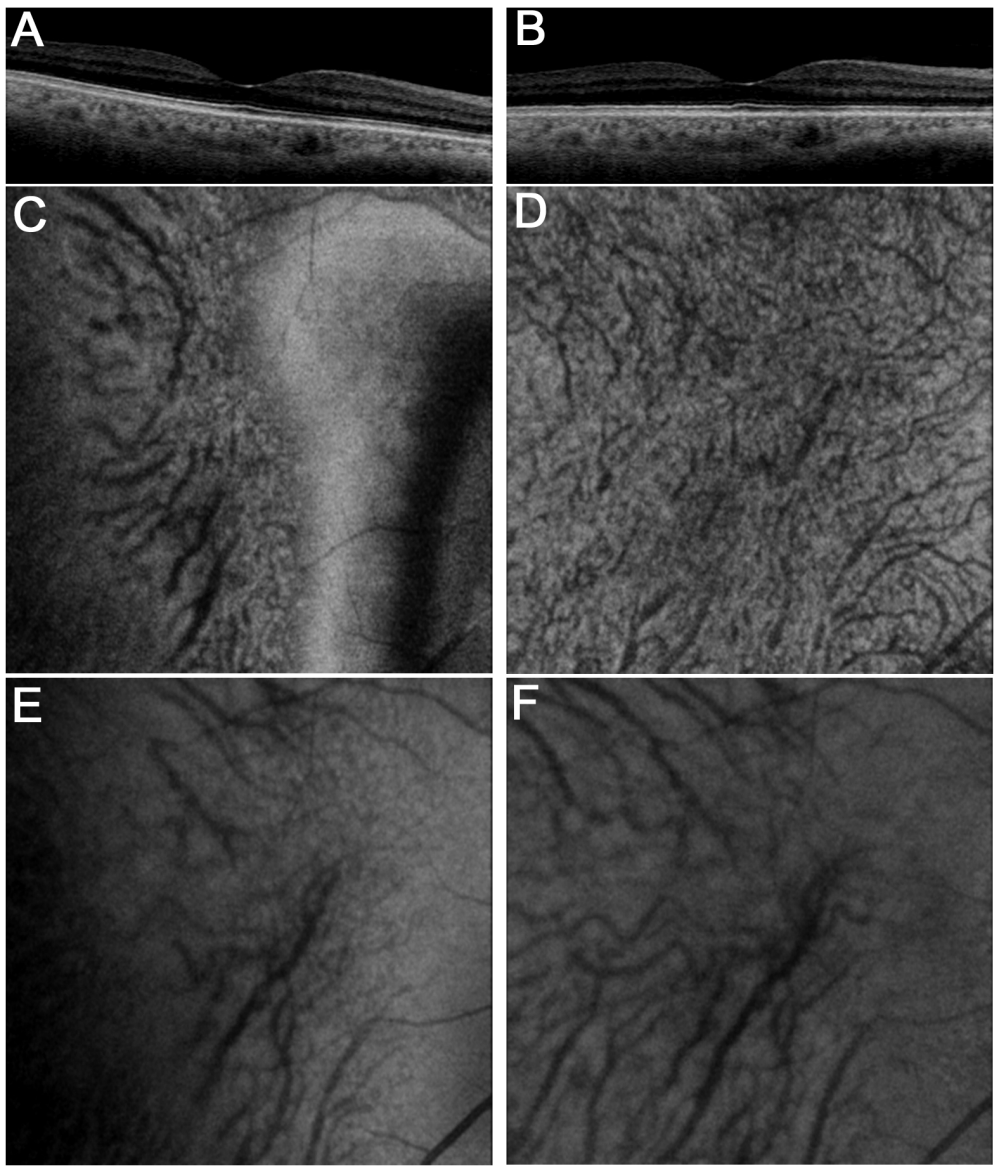
En-face images of Sattler’s and Haller’s layers after flattening of Bruch’s membrane. (A, C, E) Raw images, and (B, D, F) processed images after the flattening of chorio retinal images using Bruch’s membrane as a reference surface. En-face images after flattening at the levels of Sattler’s layer (D) and Haller’s layer (F) delineate choroidal vessels clearly, compared to en-face images before flattening (C, E).

To evaluate the morphologic characteristics in the choroidal vessels, en-face images of Sattler’s and Haller’s layers were constructed using EnView software (Topcon) which enables us to generate en-face images with the minimal thickness of 2.6 μm. Considering the horizontal choroidal layers, B-scan images were applied to the flattening function of EnView software to make the curve of chorioretinal images flattened using Bruch’s membrane as a reference surface. We manually determined the boundary between Sattler’s layer and Haller’s layer as described previously[24]. It was also reported that the mean thickness of large choroidal vessel layer corresponding to Haller’s layer and that of the medium choroidal vessel layer-choriocapillaris layer are 204.3±65.9 μm and 52.9±20.6 μm, respectively[24]. It prompted us to initially create the slab images of Haller’s layer with 79 pixels (corresponding to 205.4 μm) and those of medium choroidal vessel layer and choriocapillaris layer with 21 pixels (corresponding to 54.6 μm), and measured the diameters of large choroidal vessels. Further, the diameter of small choroidal vessels which was delineated in the inner choroidal layer alone was also quantified, because the definition of medium choroidal vessels is not clear. We at random selected three of relatively straight segments of choroidal vessels with more than 40-pixel length from individual ten nondiabetic control eyes. We measured the area and the length of the segments using freehand selection of ImageJ (National Institutes of Health, Bethesda, MD), and then divided the area by the length to calculate the mean diameter of the choroidal vascular segments. The average diameters of large and small choroidal vessels were 160.8±33.5 μm and 27.9±5.7 μm, respectively. We then selected the mean signal levels of 61 and 11 pixels along the z-axis (corresponding to 158.6 and 28.6 μm thicknesses, respectively) to generate en-face images (x-y plane) of these two layers with better vascular images. We evaluated choroidal vascular morphologies in Haller’s layer in the outer en-face images 30 pixels from the boundary between Sattler’s and Haller’s layers. The inner slab images 5 pixels from the boundary were also generated to assess the vascular changes in Sattler’s layer. These en-face images were processed further by two-dimensional Gaussian blur (1 pixel radius) using ImageJ to reduce the speckle noises. We used 1:4 as the aspect ratio to evaluate the B-scan images after the moving average was applied (Fig 1).

**Fig 2:**
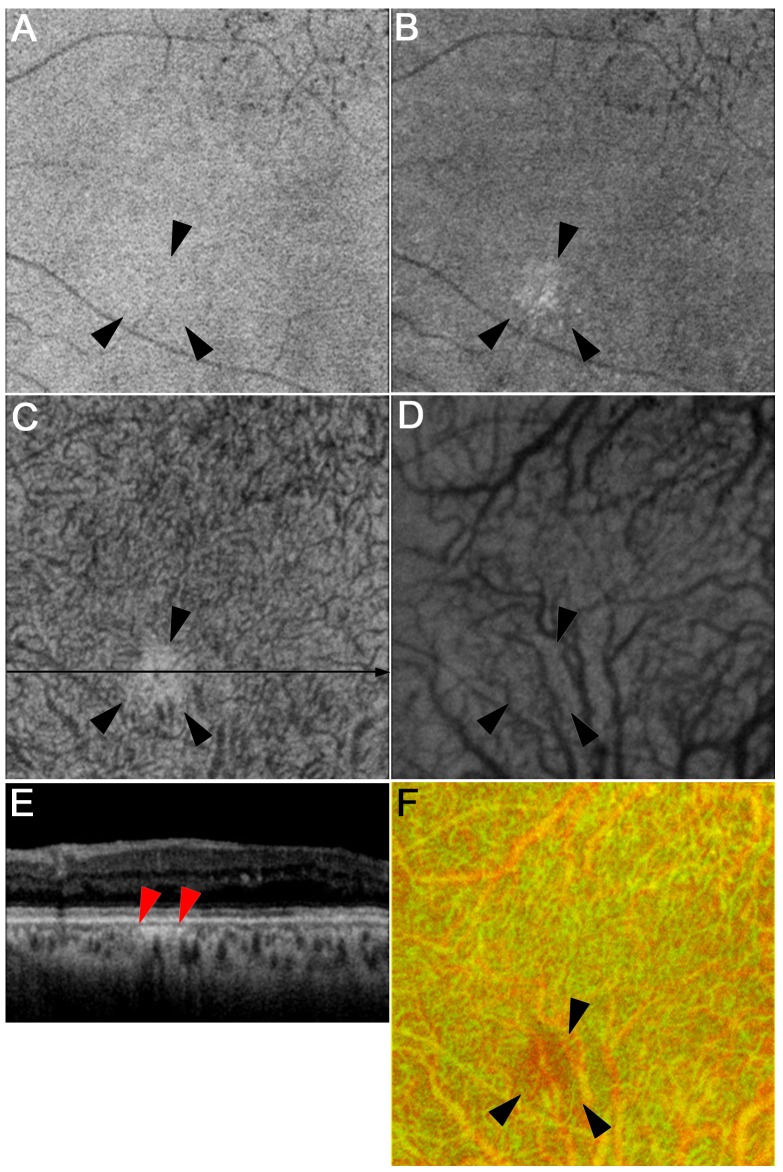
Focally unvisualized vessels in Sattler’s layer in a representative eye with moderate NPDR. The en-face images at the levels of Sattler’s layer (C) and B-scan image (E) show that the choroidal vessels are focally undelineated in Sattler’s layer (arrowheads), which is accompanied by a vascular stump at its lower edge (D, F). The en-face image at the level of the retinal pigment epithelium (A) does not show any sign in the corresponding area, although hyperreflective lesions are seen at the level of the choriocapillaris (B). (F) A merged image of Haller’s layer (red) and Sattler’s layer (green) after inverted signal levels.

**Fig 3:**
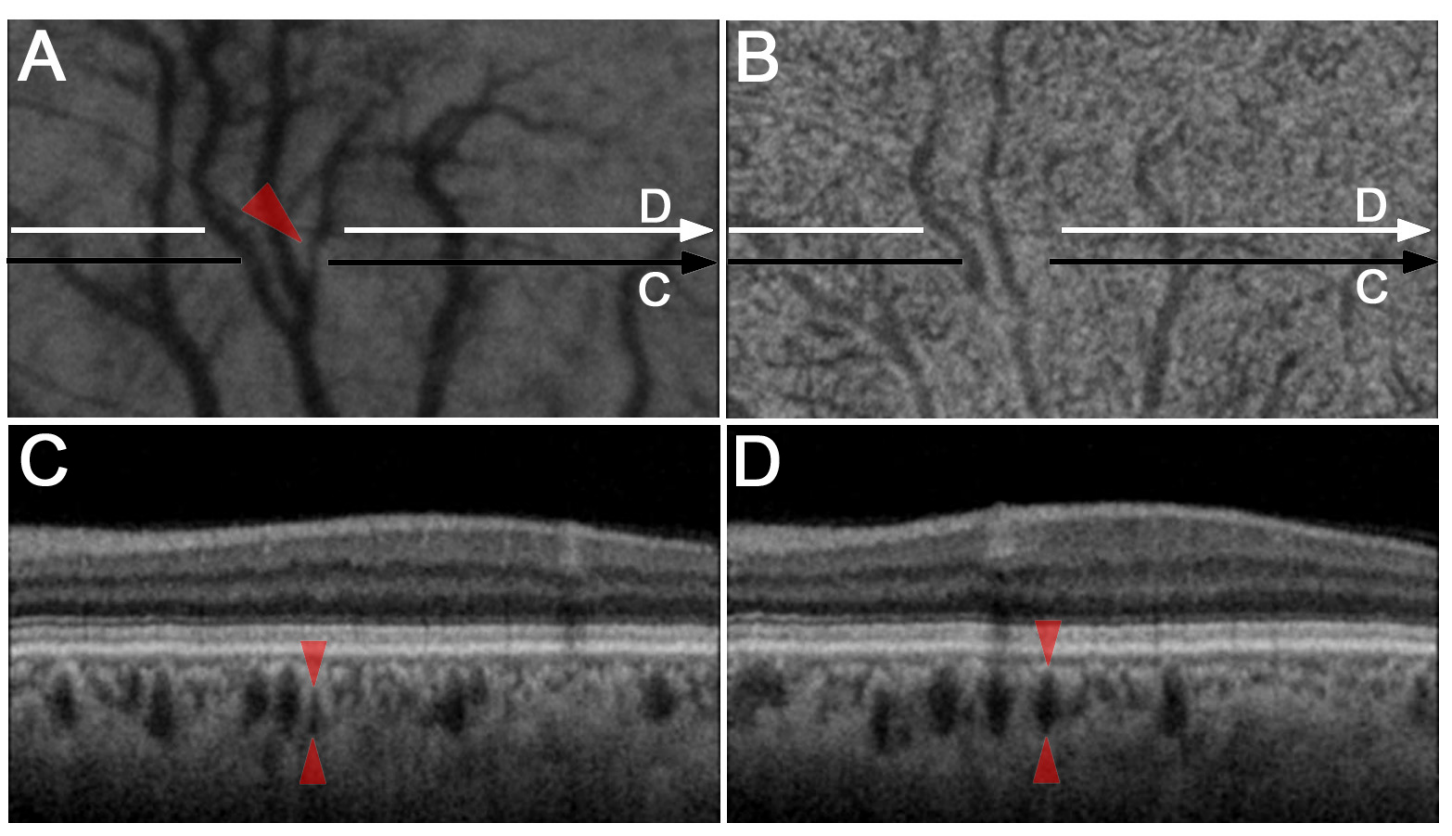
Focal vascular narrowing in Haller’s layer in an eye with mild NPDR. En-face images at the level of Haller’s layer (A) show focal narrowing of a larger choroidal vessel (arrowhead), although no definite lesions are seen at the level of Sattler’s layer (B). B-scan images show the larger vascular diameter (D, arrowheads) distal to the focal narrowing (C, arrowheads).

**Fig 4:**
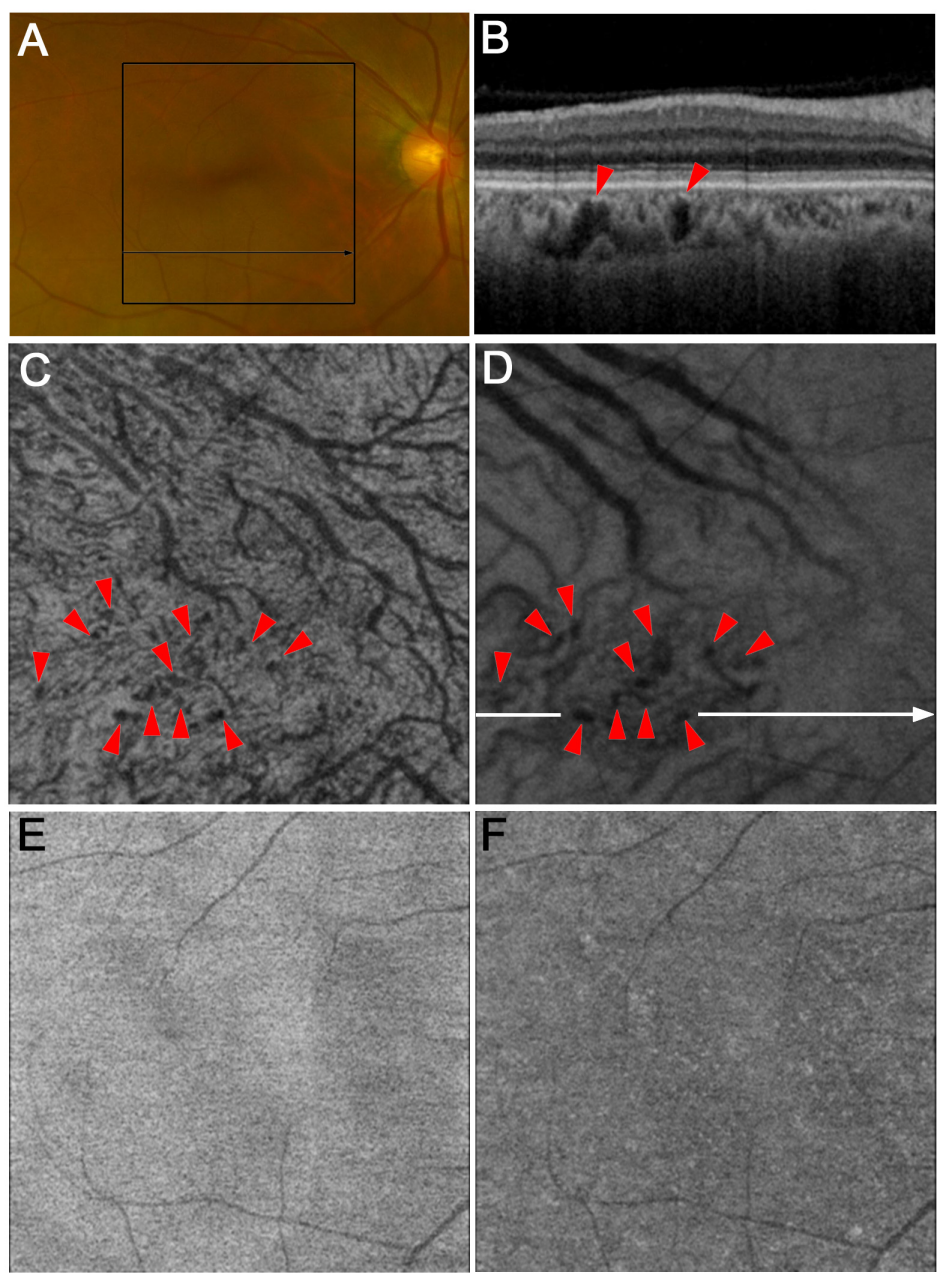
Aneurysmal changes in Haller’s layer in an eye with no apparent retinopathy. The en-face image at the level of Haller’s layer (D) shows saccular aneurysmal changes (arrowheads), which often protrude into Sattler’s layer on en-face images at the level of Sattler’s layer (C) and in a B-scan image (B, arrowheads). However, there are no definite lesions in color photography (A), or en-face images at the levels of the retinal pigment epithelium (E) and choriocapillaris (F).

**Fig 5:**
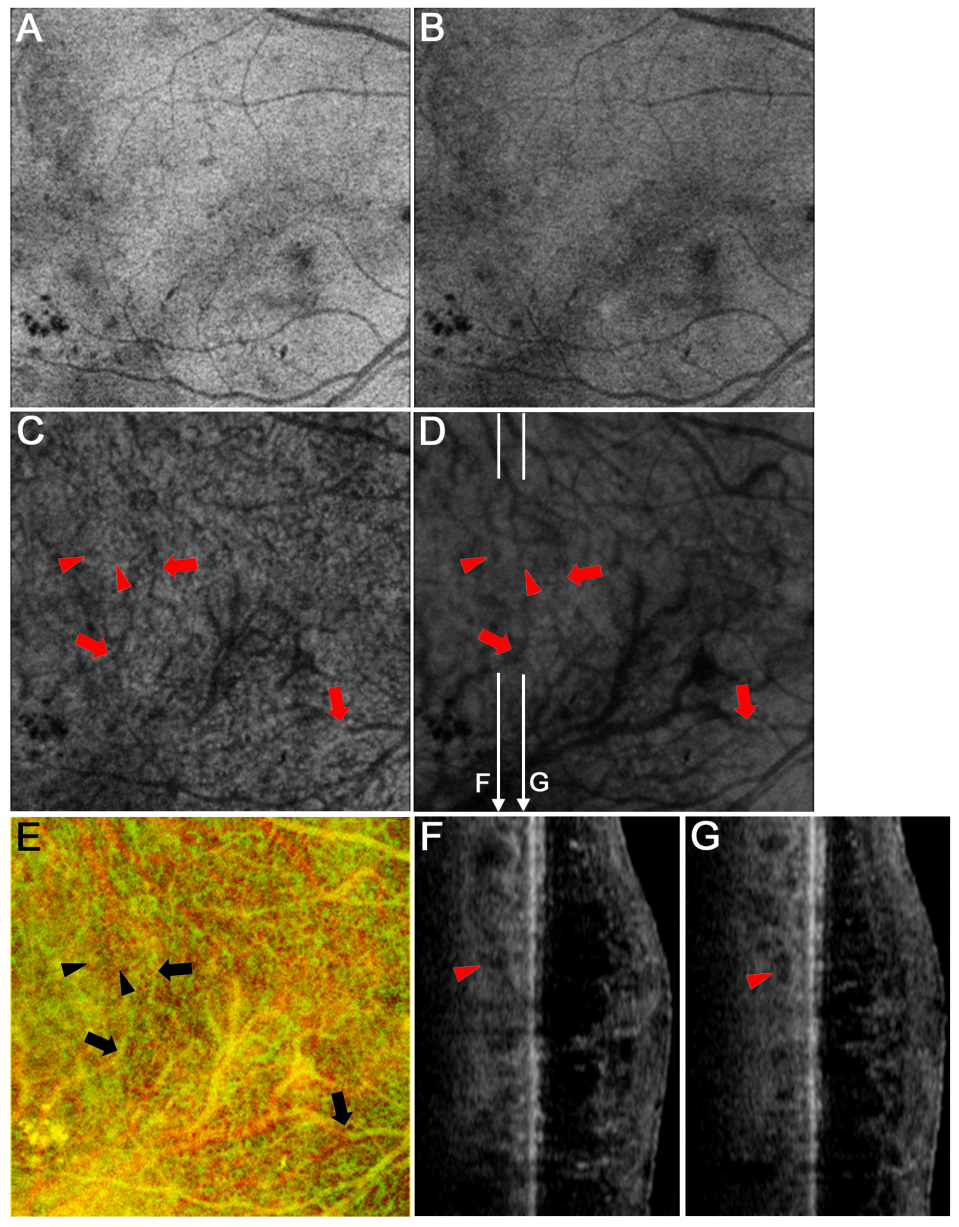
A vascular stump in Haller’s layer in a representative eye with PDR. En-face images at the levels of Sattler’s layer (C) and Haller’s layer (D) and a merged image of Sattler’s layer (green) and Haller’s layer (red) after inverted signal levels (E). (F, G) B-scan images along the white arrows in D. Most choroidal vessels in Haller’s layer connect to smaller vessels in Sattler’s layer (arrows), whereas some terminate in Haller’s layer (arrowheads). There are no definite lesions at the level of the retinal pigment epithelium (A) or the choriocapillaris (B) in the areas corresponding to the vascular stump.

We characterized the three-dimensional morphologic lesions in choroidal vessels in either Sattler’s or Haller’s layer using both B-scan and en-face images. In eyes in which Haller’s layer was thicker, we evaluated the entire volume of the layer using en-face images between the innermost and the outermost boundary in combination with B-scan images. En-face images of Sattler’s layer sometimes showed the localized areas without definite vascular structures, referred to as unvisualized vessels in Sattler’s layer (Fig 2). When we focused on the larger choroidal vessels in Haller’s layer using the outer en-face image, we often found the focally narrower vessels than their proximal and distal vessels on three-dimensional images, referred to as focal vascular narrowing in Haller’s layer (Fig 3). Larger choroidal vessels sometimes had aneurysmal changes, fusiform or saccular, most of which protruded into Sattler’s layer (Fig 4). These findings may to some extent agree with the findings in a recent publication documenting vascular remodeling in the diabetic choroid; irregular, tortuous, and beaded vessels with focal dilation and narrowing[25]. Another definite finding was the termination of larger choroidal vessels in the superficial or middle portion of Haller’s layer, referred to as the vascular stump in Haller’s layer (Fig 5). Comparison of two en-face images in Sattler’s and Haller’s layers usually showed continuity from larger vessels in Haller’s layer to smaller ones in Sattler’s layer (Fig 5). In contrast, the vascular stump in Haller’s layer showed the distal end of the choroidal vessels in the superficial or middle portion of Haller’s layer. Two retinal specialists evaluated these qualitative findings, and in the event of a disagreement, the third specialist determined the finding.

### Statistical Analysis

The results are expressed as the mean ± standard deviation. Kappa coefficient was applied to measure the inter-observer variability for qualitative findings in choroidal vasculature. The Student’s *t*-test was used to compare the quantitative data populations with normal distributions and equal variance. The data were analyzed using the Mann-Whitney *U* test for populations with non-normal distributions or unequal variance. Significant differences in the sampling distributions were determined using Fisher’s exact test or the chi-square test. *P* < 0.05 was considered significant.

## Results

### Morphologic Findings in Choroidal Vasculature on SS-OCT Images

Three-dimensional images of SS-OCT showed that of the three choroidal sublayers, and the vessels in Sattler’s layer had lower reflectivity than the surrounding stroma with a gradual decrease in their diameters from the outer to the inner side. Thirty-three (30.0%) of 110 eyes had focally unvisualized vessels in Sattler’s layer (kappa coefficient = 0.868 [95% confidence interval: 0.765–0.971]), whether the reflectivity levels of the stroma were increased or decreased (Fig 2).

Further investigation of the vascular lesions in Haller’s layer showed focal vascular narrowing in 56 eyes (50.9%; kappa coefficient = 0.764 [95% confidence interval: 0.643–0.884]; Fig 3). Some larger choroidal vessels just traversed within the 6x6-mm macular areas, and others branched off to intermediate or smaller vessels in Sattler’s layer. In contrast, choroidal vessels sometimes terminated in the superficial or middle portion of Haller’s layer, referred as to a vascular stump in Haller’s layer, in 20 eyes (18.2%; kappa coefficient = 0.878 [95% confidence interval: 0.760–0.995]; Fig 5). Such eyes had unvisualized vessels in Sattler’s layer more frequently (*P*<0.001, Table 2). Saccular or fusiform aneurysmal formations in Haller’s layer also were seen in 28 eyes (25.5%; kappa coefficient = 0.804 [95% confidence interval: 0.673–0.935]), whether at the bifurcation or not (Fig 4).

**Table 2:** Relationship between Focal Vascular Loss in Sattler’s Layer and Larger Vascular Lesions in Haller’s Layer in Patients with Diabetes.

	Presence of unvisualized vessels in Sattler’s layer	Absence of unvisualized vessels in Sattler’s layer	*P* Value
Focal vascular narrowing in Haller’s layer (present/absent)	20 eyes / 13 eyes	36 eyes / 41eyes	0.215
Vascular stump in Haller’s layer (present/absent)	18 eyes / 15 eyes	2 eyes / 75 eyes	<0.001

Diabetic eyes had focal vascular loss in Sattler’s layer, focal vascular narrowing and vascular stump in Haller’s layer more frequently than nondiabetic eyes (*P* = 0.028, *P*<0.001, and *P* = 0.026, respectively; Table 1). These three qualitative findings were not related to systemic factors including age, gender, or HbA1c, and vascular stumps in Haller’s layer alone were associated with systemic hypertension (*P* = 0.012; Table 3). We further investigated the relationship between these qualitative and quantitative parameters in the choroid and found that the subfoveal choroid was thicker in eyes with either focal vascular narrowing or a vascular stump in Haller’s layer than in eyes without these findings (*P* = 0.002 and *P* = 0.035), whereas there were no differences in the thicknesses between eyes with and without unvisualized vessels in Sattler’s layer (*P* = 0.893, Table 4).

**Table 3:** Association between Systemic Factors and Choroidal Vascular Changes in Patients with Diabetes.

		Unvisualized vessels in Sattler’s layer		Focal vascular narrowing in Haller’s layer		Vascular stump in Haller’s layer	
		present/absent	*P* Value	present/absent	*P* Value	present/absent	*P* Value
Age (years)		65.4±11.2 / 61.8±14.3	0.203	62.5±11.6 / 63.2±15.3	0.780	66.9±9.1 / 62.0±14.2	0.144
Gender	male	18/49	0.400	36/31	0.558	11/56	0.616
	female	15/28		20/23		9/34	
HbA1c (%)		7.69±1.47 / 7.61±1.36	0.825	7.71±1.45 / 7.64±1.42	0.835	7.32±1.65 / 7.75±1.37	0.294
Hypertension	present	21/45	0.675	39/27	0.051	17/49	0.012
	absent	12/32		17/27		3/41	

**Table 4:** Association between Qualitative Findings and Choroidal Thickness in Patients with Diabetes.

		logMAR VA		Retinal Thickness in the central subfield (μm)		Subfoveal Choroidal Thickness (μm)	
			*P* Value		*P* Value		*P* Value
Unvisualized vessels in Sattler’s layer	present	0.219±0.417	0.001	310±104	<0.001	243±70	0.893
	absent	0.021±0.203		250±70		245±80	
Focal vascular narrowing in Haller’s layer	present	0.147±0.356	0.016	263±81	0.573	266±69	0.002
	absent	0.012±0.200		272±91		221±78	
Vascular stump in Haller’s layer	present	0.315±0.465	<0.001	333±120	<0.001	277±68	0.035
	absent	0.028±0.216		253±70		237±77	

### Relationship between DR and Choroidal Findings

The comparative studies between DR and these choroidal findings showed that 11 eyes (32.4%) with PDR had a vascular stump in Haller’s layer, whereas this lesion was seen in only two eyes (7.4%) with no apparent retinopathy (*P* = 0.026, Table 5). Unvisualized vessels in Sattler’s layer were found in eyes with DR more frequently, but not with statistical significance (Table 5). There was no association between focal vascular narrowing in Haller’s layer and DR severity (Table 5).

**Table 5:** Choroidal Vascular Changes by Grades of Diabetic Retinopathy Severity.

	No Apparent Retinopathy (eyes)	NPDR (eyes)	PDR (eyes)	*P* Value
Unvisualized vessels in Sattler’s layer (present/absent)	4/23	17/32	12/22	0.140
Focal vascular narrowing in Haller’s layer (present/absent)	13/14	24/25	19/15	0.782
Vascular stump in Haller’s layer (present/absent)	2/25	7/42	11/23	0.026

The logarithm of the minimum angle of resolution (logMAR) VA was significantly poorer in eyes with a vascular stump in Haller’s layer and with focal vascular loss in Sattler’s layer than in eyes without these findings (Table 4). It may be compatible to the retinal thickening in the central subfield in eyes with these choroidal findings (Table 4). Eyes with focal vascular narrowing in Haller’s layer had poorer logMAR VA but not greater retinal thickness in the central subfield (Table 4).

## Discussion

In the current study, we described several morphologic changes seen on SS-OCT images in the choroidal vessels, i.e., focally unvisualized vessels in Sattler’s layer and focal narrowing, aneurysmal changes, and a stump of choroidal vessels in Haller’s layer in patients with diabetes. Diabetic eyes had unvisualized vessels in Sattler’s layer or focal narrowing or a stump in choroidal vessels in Haller’s layer more frequently than nondiabetic eyes. A vascular stump in Haller’s layer was especially associated with the DR severity, logMAR VA, and retinal thickness. Recent publications that have reported quantitative analyses of the choroid have indicated that the association between the choroidal thickness and DR or DME is controversial[13–15]. Both qualitative and quantitative findings would integratively promote an understanding of diabetic choroidopathy and its contribution to the pathogenesis of DR and DME[6].

We considered the histologic findings that corresponded to unvisualized vessels in Sattler’s layer on SS-OCT images. This finding might correspond to the focal vascular loss or small vessels with obliterated or severely narrowed lumens in Sattler’s layer. A pathohistologic publication reported focal scar or acellular nodules in inner choroidal layers including Sattler’s layer in diabetic choroidopathy[6], which might compress vasculature and concomitantly narrowed or obliterated vascular lumens. Diffuse thickening of vascular basement membrane in choriocapillaris and the deeper layers also might lead to severely narrowed lumen[6]. A few reports documented loss of capillaries in the choriocapillaris layer, which might result in the narrowing or loss of feeder or draining vessels[6, 7]. Fluorescein angiography (FA) shows delayed filling of the choriocapillaris and indocyanine green angiography (IA) showed hypofluorescent spots in the early phase[26–29]. Angiographic findings are also compatible to the speculations, although further comparative studies should be planned.

Histologic analyses showed a thickened basement membrane and arteriosclerotic lesions, which sometimes were accompanied by luminal obliteration, in choroidal arteries[6]. These findings may correspond to focal vascular narrowing or a vascular stump in Haller’s layer on SS-OCT images, and may be compatible to angiographic findings describing the disturbed perfusion as described above[6, 26–29]. However, we could not reach a definitive conclusion that these OCT findings, vascular narrowing and a stump, were sequential, because unvisualized vessels in Sattler’s layer or DR severity have associations with vascular stump but not focal narrowing (Tables 2 and 5).

SS-OCT sometimes has shown aneurysmal changes in Haller’s layer, which may be supported by histologic and angiographic reports about choroidal aneurysms in patients with diabetes[26–28, 30]. In the current study, there were no associations between aneurysmal changes and diabetes, compared to the diagnostic significance of microaneurysms in DR. Considering the diameter of larger choroidal vessels, the pathogenesis of this lesion may differ markedly from that of intraretinal microaneurysms in DR and be similar to retinal macroaneurysms.

In addition to the changes in choroidal vasculature, recent publications reported the hyperreflective lesions in the choroid[31]. SS-OCT in the current study also delineated hyperreflective foci in 11 diabetic eyes, although it remains ill-defined whether the reflective signals were modulated by the pigmentary cells including retinal pigment epithelium or not. Such lesions might correspond to lipid-laden macrophages or the precursors of hard exudates, as speculated in the diabetic retinas[32]. As another possible lesions in the histology might be focal scar or acellular nodules[6, 7]. Further study should be planned to resolve these issues.

An increasing number of publications have reported an association of the choroidal thickness with diabetes, DR, and DME[13–15, 19, 20]. We found that the choroid was thicker in eyes with focal narrowing or a stump of choroidal vessels in Haller’s layer in patients with diabetes. These choroidal lesions might be associated with vascular hyperpermeability and concomitant choroidal thickening, although further study should be planned to elucidate the relationship between angiographic and optical coherence tomographic findings[27–29]. Another possible mechanism might be that the lesions in choroidal stroma lead to choroidal thickening and compress vasculature and concomitant narrowing or obliteration of choroidal vessels[6]. The choroidal thickness has been reported to be affected by a few systemic factors[13, 22, 33–35], which also may induce vascular morphologic changes, although we could not find out the significant association of choroidal thickness with age, hypertension, or diabetes in this study (Data not shown).

We also showed that DR is associated with a vascular stump in Haller’s layer, and retinal thickness is greater in eyes with unvisualized vessels in Sattler’s layer or vascular stump in Haller’s layer. These findings might represent malnourishment in the outer retinal layers mediated via the choroidal vasculature, which concomitantly could produce VEGF and contribute to the progression of DR and DME[36]. Another possibility is that diabetes may be a common regulator that induces DR progression and promotes the pathogenesis in these choroidal vascular lesions[1, 3, 4, 6]. Eyes with these choroidal findings had poorer logMAR VA, and it remains to be elucidated how these choroidal lesions impair retinal function.

The main limitation in this study is the interpretation of vascular lumens on SS-OCT images. Retinal vessels with blood flow are accompanied with hourglass-shaped hyperreflectivities[37]. We could not conclude that the hyporeflective regions on SS-OCT images completely correspond to vascular lumens in the choroid. In addition, the image resolution of SS-OCT did not allow us to determine whether the absence of the lumens on SS-OCT images means histological vascular loss or obliteration or the severe narrowing in the choroidal vessels. In addition, the sample size is smaller in this study, and future studies of larger cohort should be planned to confirm the diagnostic or epidemiological relevance of these findings.

In the current study, we reported the morphologic findings, focally unvisualized vessels in Sattler’s layer and a vascular stump in Haller’s layer, in patients with diabetes on three-dimensional SS-OCT images. Further integrative analyses would lead to a better understanding of the pathogenesis in diabetic ocular complications.
